# The fin roundabout: Slit‐Robo and S1P signaling coordinate fin morphogenesis

**DOI:** 10.15252/embr.202255563

**Published:** 2022-07-14

**Authors:** Ivonne M Sehring, Gilbert Weidinger

**Affiliations:** ^1^ Institute of Biochemistry and Molecular Biology Ulm University Ulm Germany

**Keywords:** Cell Adhesion, Polarity & Cytoskeleton, Development, Signal Transduction

## Abstract

Development of vertebrate limbs and fins requires that tissue growth is directed outwards, away from the body. How such directed growth is achieved is a fascinating biological problem. For limb/fin formation and outgrowth, signaling between mesenchymal cells and the overlying epithelium is essential. In particular, the epithelium at the distal margin of the growing limb/fin bud, termed the apical ectodermal ridge (AER), promotes directed outgrowth of the underlying mesenchyme, e.g., by providing polarization cues for mesenchymal cell migration. Several classical signaling pathways, such as fibroblast growth factor (Fgf), hedgehog, and Wnt signaling, are involved in the regulation of the cellular events that shape the limb/fin bud (Iovine, 2007). In this issue of *EMBO Reports*, Carney and colleagues surprisingly find that the Slit‐Robo pathway, which is best known for its function in axon guidance, regulates the polarity of developing zebrafish fins (Mahabaleshwar *et al*, 2007). Intriguingly, they identify an intricate back and forth of signals between the mesenchyme and the AER. Slit ligands derived from mesenchyme act on Robo receptors in the AER to stimulate the production of sphingosine‐1‐phosphate, which then acts back on the mesenchyme to regulate cell polarity and orientation.

## Graphical abstract

A study in this issue shows that the Slit‐Robo pathway, best known for its function in axon guidance, regulates zebrafish fin polarity and morphogenesis.

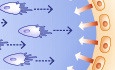

In zebrafish embryos, the early pectoral fin bud, which is homologous to amniote forelimbs, and the fold that develops into the tail fin are flat structures composed of thin ectodermal layers covering few mesenchymal cells. Transient cell–cell adhesion of lateral epidermal cells along the midline is crucial during fin outgrowth, and several mutants have been identified in forward genetic screens that cause blistering (Carney *et al*, 2010). The genes affected in blistering mutants known so far code for extracellular matrix proteins. In this issue of *EMBO Reports*, Mahabaleshwar *et al* (2022) characterize another blistering mutant termed *stomp* and surprisingly find that it represents a hypomorphic loss‐of‐function mutation of the secreted axon guidance protein Slit3. Slits are secreted glycoproteins that function as the main ligands of Roundabout (Robo) receptors. The Slit‐Robo signaling axis was first described as an essential regulator of axon path finding and neuronal migration. Since then, it was shown that Slit‐Robo signaling is involved in the regulation of various additional developmental processes such as migration of other cell types, cell proliferation, angiogenesis, and vascularization (Blockus & Chédotal, 2016). In a thorough series of genetic studies including the creation of maternal zygotic *slit3* mutants and double mutants with another Slit ligand, *slit1a*, Carney and colleagues firmly establish that the Slit proteins act partly redundantly in preventing blistering. They show that Slit proteins are expressed in the fin mesenchyme, while all three zebrafish *robo* receptor genes are expressed in the AER. Importantly, morpholino‐based knockdown and TALEN‐mediated knock out of *robo1* also resulted in blistering.

To identify mediators acting downstream of Slit‐Robo signaling, the authors looked at other mutants with fin blistering phenotypes. Expression of the above‐mentioned genes for extracellular proteins shown to be associated with blistering was not affected in *slit3* mutants, but the authors found a synergistic effect with the *miles apart* mutant. In this mutant, the sphingosine‐1‐phosphate receptor 2 (*s1pr2*) is affected, suggesting a new link between Slit‐Robo and sphingosine‐1‐phosphate (S1P) signaling during fin development. S1P is a sphingolipid metabolite that acts through a family of receptors to regulate such divergent processes as cell migration, growth, and survival (Maceyka *et al*, 2012). In the fin fold, an interesting complementary expression pattern can be observed: The authors show that S1P is co‐expressed with Robo receptors in the AER, while S1pr2 is expressed in the Slit ligand‐expressing mesenchyme (Fig 1). Using a variety of genetic and biochemical assays, they analyzed the interaction between the two signaling pathways and show that Slit‐Robo acts upstream of S1P, by promoting the generation or release of S1P.

**Figure 1:**
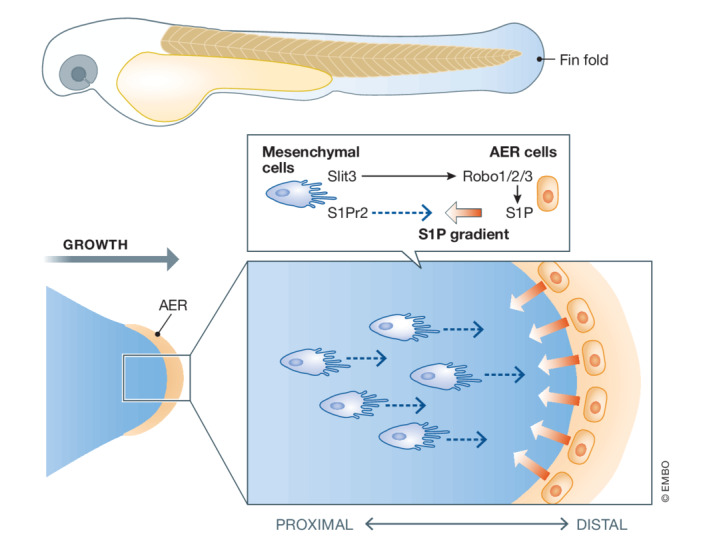
Slit‐Robo and S1P signaling during zebrafish fin‐fold morphogenesis Mesenchymal cells of the fin bud generate Slit ligands (Slit), which act on Robo receptors (Robo 1/2/3) in apical ectodermal ridge (AER) cells. Robo signaling stimulates the production and/or release of sphingosine‐1‐phosphate (S1P) from AER cells, resulting in a presumptive gradient. This self‐generated gradient acts back on mesenchymal cells, which express the S1P receptor S1Pr2, to regulate polarity and migration.

Why do disturbances in these signaling interactions result in blistering? Using high‐resolution live imaging, the authors analyzed the migration of mesenchymal cells towards the AER. These cells display a polarized morphology with filopodia directed towards the fin tip. In both *slit3* and *slpr2* mutants, polarization and elongation are impaired, and cells fail to properly migrate towards the AER. It was previously shown that signaling from sphingosine‐1‐phosphate receptors regulates cell–fibronectin interaction, and through the activation of G‐Proteins, affects cytoskeletal dynamics, and induces the formation of stress fibers (Yamamura *et al*, 2000; Matsui *et al*, 2007). Concordantly, the authors show that in both *slit3* and *s1pr2* mutants, focal adhesions and stress fibers are reduced in mesenchymal cells.

Based on their findings, the authors propose a model in which the activation of S1P via Slit‐Robo signaling is important for the maintenance of adhesion between mesenchymal cells and epidermal sheets of the fin fold. Furthermore, a presumed gradient of S1P signal (due to the localized production in the AER) results in a fine‐tuned progression of adhesiveness along the fin fold, which appears to regulate the directed migration of the mesenchymal cells.

In this elegant work, the authors define a novel pathway regulating morphogenesis and directed growth of zebrafish fins. While experimental proof for an S1P gradient is still lacking, their findings open interesting new avenues for future research. It will for example be important to test to which extent this signaling axis is conserved in mammalian limb development. Furthermore, the downstream modulators between Robo receptors and S1P activation are so far not known. Robo receptors do not possess autocatalytic or enzymatic activity, and several signaling molecules mediate downstream signaling progression. The authors have tested some known mediators, which, however, seem not to be involved in establishing the Robo‐S1P axis. Importantly, their work provides a new paradigm for signals that can mediate Slit‐Robo signaling, and it will be intriguing to learn whether sphingosine‐1‐phosphate acts downstream of this important pathway also in other systems.
